# Photoelasticity-based evaluation of cellular contractile force for phenotypic discrimination of vascular smooth muscle cells

**DOI:** 10.1038/s41598-019-40578-7

**Published:** 2019-03-08

**Authors:** Shukei Sugita, Eri Mizutani, Masatoshi Hozaki, Masanori Nakamura, Takeo Matsumoto

**Affiliations:** 1 Department of Electrical and Mechanical Engineering, Graduate School of Engineering, Nagoya Institute of Technology, Nagoya, Japan; 2 Department of Techno-Business Administration, Graduate School of Engineering, Nagoya Institute of Technology, Nagoya, Japan; 3 Department of Mechanical Systems Engineering, Graduate School of Engineering, Nagoya University (Present), Nagoya, Japan

## Abstract

Vascular smooth muscle cells (VSMCs) have two distinct phenotypes: contractile and synthetic. The major difference between these phenotypes lies in the magnitude of the contractile force produced by the cell. Although traction force microscopy (TFM) is often used to evaluate cellular contractile force, this method requires complex preprocessing and a sufficiently compliant substrate. To evaluate the contractile force and the phenotype of living VSMCs with minimal effort and in a manner independent of the substrate stiffness, we propose a photoelasticity-based method using retardation, which is related to the difference between the first and second principal stresses and their orientation. The results demonstrate that actin filaments co-localize with areas of high retardation in cells, indicating that the retardation of VSMCs is promoted by actin filaments. The retardation of cells treated with calyculin A and Y-27632 tended to be larger and smaller, respectively, than that of control cells. Cell traction force significantly correlates with total cell retardation (*r*^2^ = 0.38). The retardation of contractile VSMCs (passage 2) was significantly higher than that of synthetic VSMCs (passage 12). These results indicate that cell retardation can be used to assess cell contractile force and, thus, determine the phenotype of VSMCs.

## Introduction

Some cells generate a contractile force through stress fibres (SFs), composed of actin and myosin filaments, by sliding bundles of myosin filaments over bundles of actin filaments. The contractile forces strongly influence fundamental functions of cells, such as migration^1,2^, mechano-sensing^3^, and deformation^4^.

Vascular smooth muscle cells (VSMCs) are found in two distinct phenotypes: contractile and synthetic^5^. Contractile VSMCs produce more contractile force^6^ and are found in healthy arteries^7^. Synthetic VSMCs exhibit higher growth rates and migratory activity^8,9^ and contain many organelles^8^, producing more proteins^10^. Synthetic VSMCs are found in arteries with atherosclerosis^11^, hypertension^12^, and aneurysms^13^, suggesting that this VSMC phenotype is indicative of changes in arterial health.

Cell contractile force is evaluated from cell traction force (CTF), which is a tangential tension exerted by cells on the extracellular matrix or the underlying layer. Traction force microscopy (TFM)^14^ is a representative method for determining the CTF exerted by a cell on a substrate surface. In TFM, microspheres are embedded in a compliant substrate, and their displacement by cell contraction is used to measure the strain and thus determine the cell CTF. Other methods to assess CTF include quantification of the deflection of bent micro-pillars^15^ and wrinkles of the substrate on which cells are seeded^16^. The main drawback of TFM and the methods evaluating micro-pillar deformation and substrate wrinkles is that cells must be sparsely seeded and the substrate must be compliant enough to allow large deformation. In our experience, it is challenging to perform TFM measurements for a substrate with a Young’s modulus larger than 100 kPa because the displacements of microspheres are minute in these substrates. In nature, VSMCs are embedded in extracellular matrix, which is far stiffer (~1 MPa)^17^. Because the extracellular matrix stiffness influences VSMC phenotype^18,19^, the phenotype of cells cultured on a soft substrate are likely to differ from those of cells in natural conditions.

To evaluate the contractility and thus phenotype of living VSMCs on substrates of any stiffness, we propose a photoelasticity-based method. In theory, when a force is applied to photoelastic materials, their refractive indices change due to a conformational change in molecular structure. When two rays of light travel at different speeds through a material, the slow ray lags behind the fast ray while passing through the material. The distance represented by this lag is called retardation. Retardation is related to the difference in the indices of refraction for the slow and fast rays or the state of the stress at that point. Under the assumption that the refractive index stays constant, retardation measurements provide information on the mechanical state.

The aim of the present study is threefold. First, we look at identifying the subcellular component that determines the overall retardation in VSMCs, particularly cytoskeletal components, namely, actin fibres, microtubules, and intermediate filaments. Second, we examine whether changes in contractile forces in VSMCs are reflected in changes in retardation. Finally, we evaluate whether the VSMC phenotype can be determined from the retardation.

## Results

### Retardation originating from actin filaments in VSMCs

**Figure 1:**
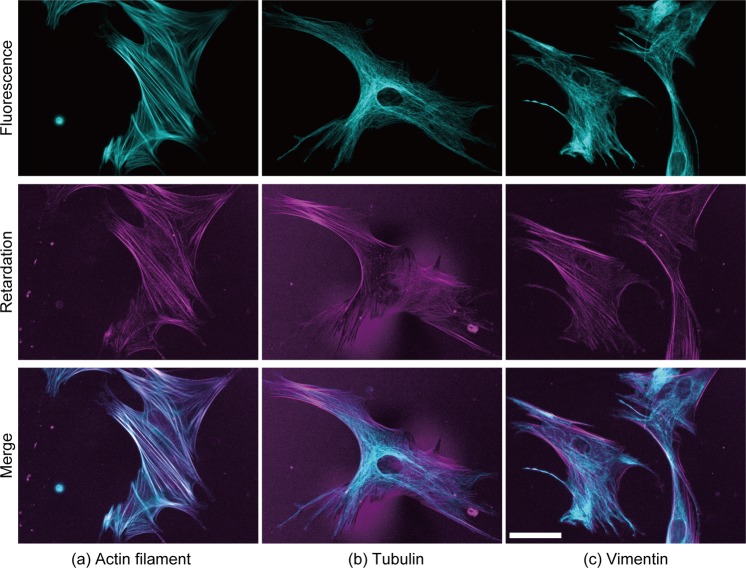
Typical images of fluorescently labelled cytoskeletons and the retardation of VSMCs. Three major cytoskeletal components, *i*.*e*., (**a**) actin, (**b**) β-tubulin, and (**c**) vimentin, were stained with fluorescent dyes in VSMCs. The fluorescence images shown in cyan (top row) and the retardation images shown in magenta (middle row) are merged in the bottom row. White colours in the merged images show the co-localization between the cytoskeleton and retardation. Bar in the bottom right panel = 50 µm and applies to all images.

In a preliminary observation, retardation images of VSMCs seemed to show a cytoskeletal network structure. While actin filaments have been observed in retardation images^20^, spindles in sea urchin, which are composed mainly of microtubules, have also been observed in retardation images^21,22^. Thus, to evaluate which cytoskeletal component in VSMCs mainly generates retardation, three major cytoskeletal components, namely, actin filaments, microtubules, and intermediate filaments, were labelled with fluorescent phalloidin, tubulin antibody, and vimentin antibody, respectively. Figure 1 compares images of fluorescently labelled cytoskeletons with their retardation images. Actin filaments were well co-localized with high retardation areas (Fig. 1a), whereas microtubules (Fig. 1b) and intermediate filaments were not (Fig. 1c). Thus, the main source of retardation was actin filament bundles or SFs in VSMCs.

### Retardation of VSMCs changes when their contraction force is regulated

**Figure 2:**
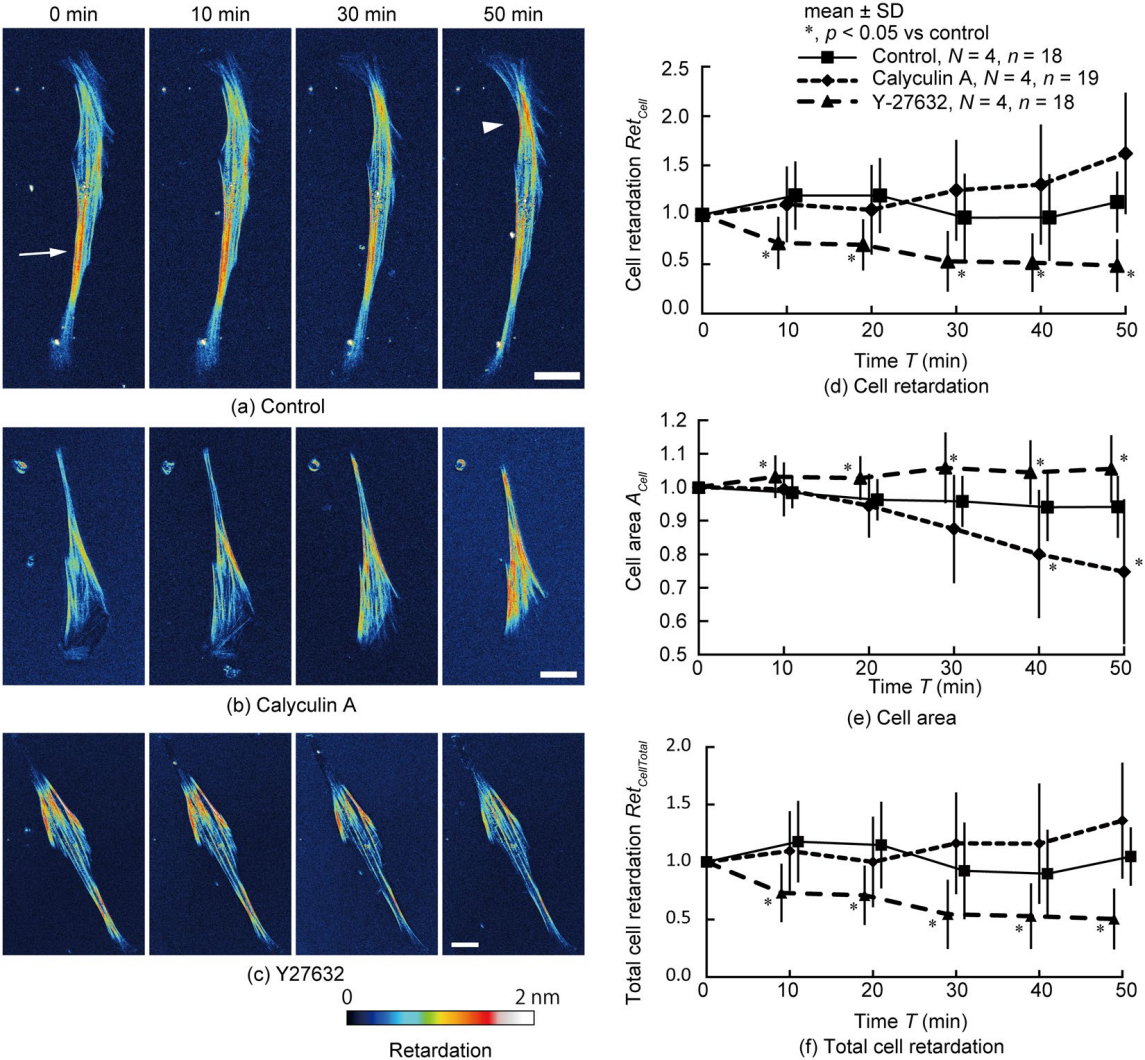
Changes in retardation with time when the chemical reagent was applied to the cell to alter the cellular contraction state. (**a**–**c**) Typical time-lapse images of retardation of single cells after application of (**a**) DMEM (control), (**b**) calyculin A, and (**c**) Y-27632 solution. Bars = 30 µm. The arrow and the arrow head in (**a**) show the high retardation area. (**d**–**f**) Changes in (**d**) cell retardation, *Ret*_*Cell*_; (**e**) cell area, *A*_*Cell*_; and (**f**) total cell retardation, *Ret*_*CellTotal*_, with time. Data in (**d**–**f**) were normalized by the data at 0 min. *p < 0.05 vs control; *N*, Number of dishes; *n*, Number of cells. The colour bar of retardation is applied to all images.

Second, we examined whether changes in contractile forces in VSMCs are reflected by changes in retardation. Calyculin A and Y-27632 were applied to VSMCs to stimulation VSMC contraction and relaxation, respectively, and changes in retardation with time were examined. Figure 2a,c shows typical time-lapse images of retardation in single cells. Even in control cells (Fig. 2a, Movie 1), retardation changed with time. In cells treated with calyculin A, retardation around the centre of the cell area increased (Fig. 2b, Movie 2). In contrast, treatment with Y-27632 resulted in decreased retardation (Fig. 2c, Movie 3). Figure 2d shows the time course of the change in cell retardation, *Ret*_*Cell*_, per pixel within the cell. Upon treatment with calyculin A, the retardation gradually increased, and at 50 min, the retardation had increased 1.6-fold compared to the value at 0 min, although this increase was nonsignificant compared to the control cells (*p* = 0.07). Treatment with Y-27632 decreased the cell retardation; the cell retardation was significantly lower than that in the control cells beginning at 10 min and almost halved after 50 min. These results indicate that cell retardation becomes larger with an increase in cell contraction force and vice versa.

Reportedly, the cell area was decreased^23^ and increased^24^ by calyculin A and Y-27632, respectively. Thus, we hypothesized that the increase in cell retardation, *Ret*_*Cell*_, induced by calyculin A treatment can be attributed to an increase in the number of SFs in the cell height direction due to a decrease in the cell area. As Fig. 2e shows, we evaluated changes in the projected area of cells, *A*_*Cell*_. The projected area of the cells 40 min after calyculin A application was significantly smaller than that of the control. At each time point, the projected area of the cells treated with Y-27632 was significantly higher than that of control cells. To consider the changes in cell area due to the administration of contraction and relaxation reagents, we evaluated the total cell retardation, *Ret*_*CellTotal*_, by multiplying the cell retardation, *Ret*_*Cell*_, by the cell area, *A*_*Cell*_. Figure 2f shows the time course of the total cell retardation, *Ret*_*CellTotal*_. The total cell retardation of calyculin A-treated cells still tended to be higher than that of the control cells at 50 min, although this difference was nonsignificant (*p* = 0.12), and the value of for Y-27632-treated cells was significantly smaller. These results demonstrate that cell retardation, *Ret*_*Cell*_, is changed by calyculin A and Y-27632 treatments, even when changes in cell projected area are taken into account.

### Cell traction force is proportional to cell retardation

**Figure 3:**
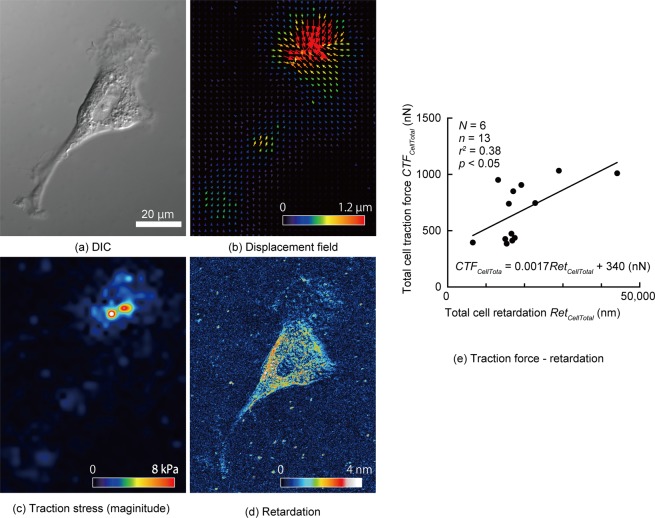
Relationship between cell traction force (CTF) and retardation. (**a**–**f**) Typical images of (**a**) differential interference contrast imaging, (**b**) displacement field of beads embedded in the polyacrylamide gel, (**c**) magnitude of traction stress, and (**d**) retardation of a VSMC. (**e**) Relationship between the total cell traction force, *CTF*_*CellTotal*_, and the total cell retardation *Ret*_*CellTotal*_, of VSMCs; *N*, Number of dishes; *n*, Number of cells.

TFM^14^ provides the spatial distribution of the cell traction stress that results from a balance of forces exerted by a cell via focal adhesions. In contrast, retardation measurement gives the distribution of stress generated in intracellular structures. Thus, a direct comparison of the cell traction stress with the retardation is not appropriate. To compare the quantity that represents the whole cell, we used CTF, a standard measure of the cell traction stress in single cells, in conjunction with total cell retardation. Figure 3 shows (a) differential interference contrast (DIC), (b) displacement field, (c) traction stress and (d) retardation images of a VSMC on a Polyacrylamide (PAA) gel with a Young’s modulus of 16 kPa. The total cell traction force was 0.67 ± 0.26 µN (mean ± SD, *n* = 13). Figure 3e plots the CTF obtained from the traction stress of each cell against its total cell retardation, *Ret*_*CellTotal*_. Here, we demonstrate that the CTF correlates significantly with the total cell retardation (*r*^2^ = 0.38).

### Retardation of VSMC changes based on phenotype

**Figure 4:**
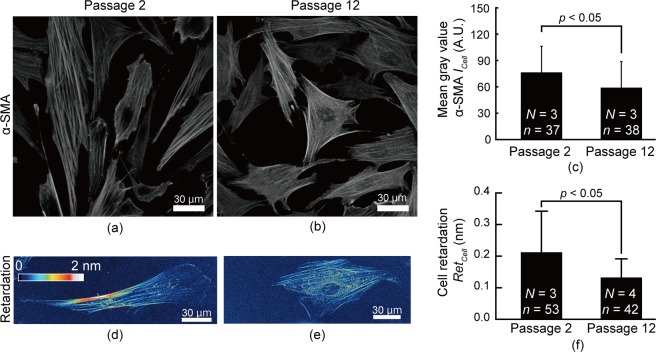
Differences in retardation between VSMCs at passages 2 and 12. (**a**–**c**) Typical images of fluorescently labelled α-SMA of (**a**) passage 2 and (**b**) passage 12 VSMCs and their mean intensity (**c**). (**d**–**f**) Typical images of retardation of (**d**) passage 2 and (**e**) passage 12 VSMCs and (**f**) their cell retardation, *Ret*_*Cell*_. *N*, Number of dishes; *n*, Number of cells.

A phenotypic change between passages 2 and 12 of porcine VSMCs was detected with fluorescently labelled α-smooth muscle actin (*α*-SMA)^25^. Fluorescent images showed that low passage cells (Fig. 4a) had more developed SFs than did high passage cells (Fig. 4b). The average intensity of *α*-SMA in low passage cells was significantly higher than that in high passage cells (Fig. 4c), indicating that low passage cells have a more contractile phenotype. In looking at retardation images, we found a higher level of retardation in low passage VSMCs (Fig. 4d) than in high passage cells (Fig. 4e) and noted a significant difference between low and high passage VSMCs (Fig. 4f). These results indicate that VSMCs with more of a contractile phenotype have higher retardation.

### Retardation is higher in the central region of a cell

**Figure 5:**
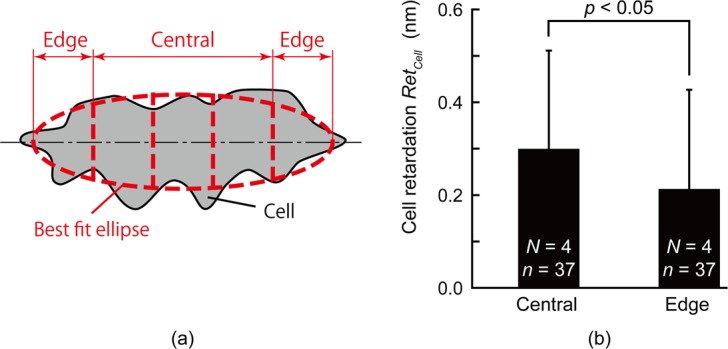
Distribution of cell retardation, *Ret*_*Cell*_, of VSMCs. (**a**) Schematic illustration of the way to divide cells into central and edge regions. (**b**) Comparison of cell retardation, *Ret*_*Cell*_, of VSMCs between the central and edge regions in single cells. *N*, Number of dishes; *n*, Number of cells.

Retardation within a cell is not spatially uniform, as shown in Figs 2 and 3. Figure 5 compares the average retardation in the central region of a VSMC to that of a region at the edge (see *Image Analysis* for definition of the central and edge regions). The retardation in the central region (0.18 ± 0.10 nm) is significantly larger than that in the edge region (0.11 ± 0.08 nm).

## Discussion

This study demonstrates that the retardation of VSMCs is correlated with their contractility. Cell retardation is well co-localized with SFs, which are considered to generate the contraction force of VSMCs. Calyculin A treatment increased the cell retardation of VSMCs, whereas Y-27632 treatment decreased cell retardation. Although calyculin A- and Y-27632-treated cells underwent cell area changes, as reported previously^23,24^, a difference in cell retardation remained evident after the correction taking into account the cell area change. The cell traction force of VSMCs obtained from conventional TFM correlates significantly with the total cell retardation. The cell retardation of VSMCs at passage 2, which are considered to be of the contractile phenotype, was significantly higher than that of VSMCs at passage 12, which are considered to exhibit the synthetic phenotype. Taken together, these results show that retardation can be used to evaluate the contractility of VSMCs and determine their phenotype.

Evaluation of cell contractility using cell retardation has several advantages. First, retardation measurement enables evaluation of the contractility of cells in contact with each other, which cannot be achieved with TFM. The retardation measurement will help provide information about mechano-transduction through mechanical cell-cell interactions. Second, retardation measurements helps in understanding the spatial distribution of contraction force inside cells. As Fig. 2a shows, the retardation is not uniform, even in control cells. Such a retardation distribution will provide new insights into cell mechanobiology. Third, fixation and staining are not required, allowing this label-free method to be used for live imaging of contractility, as in Fig. 2. Furthermore, there is no restriction on the stiffness of the substrate in this method, meaning that the retardation measurement provides an opportunity to study parameters of cell physiology, such as cell contractility, on substrates with a stiffness similar to that of the *in vivo* state.

Retardation measurement enables the evaluation of the phenotype of VSMCs based on their contractility. Because phenotypic changes of VSMCs are found in vascular diseases, such as atherosclerosis^11^, hypertension^12^, and aneurysms^13^, measurement of retardation could be useful for evaluating the pathophysiological state of VSMCs. To date, the phenotype of VSMCs has been confirmed by α-SMA^25^, SM22α^26^, and calponin^26^ staining, a process that requires fixation. Fixation not only kills cells but also constitutes a time-consuming and expensive process. As an alternative to staining phenotype markers, contractile forces can be assessed in living cells using a FRET-based actinin tension sensor^27^ method. In this method, deformation of actinin, which diagonally bundles actin filaments, is evaluated. The results are therefore affected by the bundling angle of actinin. To the best of our knowledge, it remains unclear how variable the bundling angle is. The results also change if there is a shear between the bundles of actin filaments. These points make precise calibration of the FRET-based actinin tension sensor method difficult. Compared to those methods, our method is capable of easily and quantitatively evaluating the contractility and phenotype of living VSMCs. Furthermore, retardation allows single cell analysis in a pool of cells to identify cells with a phenotype of interest. In the future, cell retardation measurement might be used to evaluate pharmacological effects on cells.

Retardation is increased by the contraction of SFs, as shown in Fig. 2b. The increase in contraction might be caused by an increase in the amount of myosin intercalated with actin. Application of calyculin A induces contraction of SFs in smooth muscle^28^ and increases CTF^29^. Interestingly, Peterson *et al*.^29^ reported that SFs were more stretched in central regions than in peripheral regions when calyculin A was applied. Figure 2b of the present study shows that retardation increased mainly in the central region upon calyculin A application, implying that retardation is increased by SF contraction. According to Matsushita *et al*.^30^, when tension is applied to an actin filament, the twist angle of the filament is decreased, demonstrating a structural change in the actin filament. This result indicates that the structural change in actin filaments is attributable to SF contraction, leading to an optical property variation in actin filaments and, in turn, increased cell retardation.

There are other possible mechanisms for the change in retardation of cells. The first possible cause is the formation of SFs. Application of calyculin A induces the formation of SFs in Swiss 3T3 cells^29^. Since retardation is proportional to the volume of birefringent material^31^, an increase in the amount of SFs resulting from polymerization of globular actin might cause an increase in retardation. According to Katoh *et al*., the bundles of actin filaments in SFs appeared to be twisted^32^. Stretching of the SFs would align the bundles of actin filaments in the stretched direction. An increase in the alignment consistency of actin filaments might also have caused an increase in the retardation. When alignment of birefringent materials increases along the slow axis, the retardation increased^33^. Thus, the retardation of VSMCs might have increased due to the alignment of actin filaments, although it remains unclear whether calyculin A aligns actin filaments. Future studies should examine these possibilities.

Even in control VSMCs treated with Dulbecco’s modified Eagle’s medium (DMEM) solution, the spatial distribution of retardation within the cell changes with time: high retardation is observed in the left bottom area (arrow) at 0 min and in the right top area (arrow head) at 50 min (Fig. 2a), although the average retardation remains almost the same at 50 min, as shown in Fig. 2d. Generally, SFs in cells dynamically change their position and structure on this time scale. Thus, the local force in SFs might have changed heterogeneously in control VSMCs, resulting in changes in the distribution of cell retardation.

The CTF obtained with TFM was higher in the edge region of cells (Fig. 3c), whereas the retardation was higher in the central region (Fig. 3d). This difference can be explained as follows. Stress measured with TFM consists of the stress in the gel or outside the cells and is generated by cells pulling the gel through focal adhesion sites that are mainly located at cell edge regions *in vitro*. In contrast, retardation originates from SFs within cells. Thus, the CTF obtained with TFM is different from the CTF obtained with retardation.

Although high retardation regions coincided with SFs for the sample on a glass-bottom dish (Figs 1a and 2a–c), this was not the case for the sample on the PAA gel (Fig. 3d). This means that the retardation pattern is affected by the substrate. As shown in many studies^18,19,34–36^, the cell condition varies substantially depending on the substrate stiffness. Fibroblasts cultured on flexible substrates have finer microfilaments in SFs^37^. VSMCs cultured on PAA gels had fewer SFs than those cultured on glass-bottom dishes (see Supplementary Information S1). As a consequence, the retardation of SFs on PAA gels was considered to be lower than that on glass-bottom dishes, although high retardation was found along the SF structure, as shown in Fig. S1c,d. In looking at Fig. S1c,d, however, we found high retardation in the non-SF area and low retardation in the SF structure. These results suggest that the PAA gel added noise to the retardation measurement of SFs. As shown in Supplementary Information S2, the PAA gel is a birefringent material. Moreover, the retardation increases in accordance with stretch. When a cell is cultured on a PAA gel, the gel is pulled in various directions by the cell. Consequently, the retardation pattern on the PAA gel is determined by the interaction between cell retardation and gel retardation. Taking these results together, we conclude that there are two possible reasons for the result that high retardation regions did not always coincide with SFs on a PAA gels: (1) SFs on the PAA gels did not develop as well as those on the glass-bottom dishes and (2) PAA gels themselves have retardation and affect the retardation measurement in the cell area.

This study demonstrates that retardation measurement is useful for evaluating the cell phenotype in VSMCs. However, this method would be difficult to use in cells within tissues because these tissues contain collagen fibres, which have high retardation in nature^31^. According to our previous study^31^, the retardation of aortic tissue with a thickness of 100 µm is approximately 30 nm. This value is larger than the cell retardation measured in the current study, approximately 0.3 nm (Fig. 4h), implying that changes in cell retardation would not be detected in aortic tissue. Thus, retardation measurement can only be applied to VSMCs cultured *in vitro*.

In conclusion, SFs represent the main source of retardation in VSMCs. Cell retardation increases and decreases when VSMCs contract and relax, respectively, and correlates significantly with CTF. Therefore, retardation is a good indicator of the contractility of VSMCs. VSMCs with the contractile phenotype show a higher retardation than those with the synthetic phenotype, suggesting that retardation can be used to evaluate VSMC phenotype.

## Methods

### Cell preparation

Porcine aortic tissues were obtained from a local slaughterhouse. VSMCs were isolated from porcine aortic tissues using an explant method described in detail by Nagayama *et al*.^38^. The cells were cultured in Dulbecco’s modified Eagle’s medium (DMEM) supplemented with 10% foetal bovine serum (S1820, Biowest) and 1% penicillin-streptomycin at 37°C in 5% carbon dioxide and 95% air.

Retardations of passage 2 and 12 cells were compared because lower passage VSMCs have a contractile phenotype, while higher passage VSMCs dedifferentiate towards the synthetic phenotype^39^.

### Retardation measurement

The VSMCs were incubated on glass-bottom culture dishes (No. 1, Matsunami Glass Industry) coated with 5% fibronectin (F1141, Sigma-Aldrich) in phosphate-buffered saline (PBS) for 60 min. The cells were imaged under an inverted fluorescence microscope (IX71, Olympus) equipped with a birefringence imaging system (Abrio-LS, CRi)^21,40^ to analyse the retardation in samples. Because the retardation in background areas changed with time, a background image was taken just before imaging a cell. Cell retardation images were processed by subtracting background retardation from cell retardation. The retardation image was captured through an objective lens of 60× (UPLSAPO60XW, NA = 1.20, Olympus; UPLSAPO60XS2, NA = 1.30, Olympus) or 30× (UPLSAPO30XS, NA = 1.05, Olympus) with silicone immersion oil (SIL300CS-30CC, Olympus). DIC images of the cells were also obtained.

### Immunofluorescence microscopy

VSMCs were seeded on a glass-bottom culture dish coated with fibronectin. After incubation for 18–24 h, the cells were fixed with 10% neutral buffered formalin, treated with 0.2% Triton X-100 for 5 min, and blocked with 2 or 3% bovine serum albumin solution for 30 min. The cells were then stained with fluorescent probes. For F-actin staining, the cells were immersed in 6.6 µM fluorescently labelled phalloidin (Alexa-Fluor 488 Phalloidin, A12379, Life Technologies) for 20 min. For vimentin and microtubule staining, the cells were immersed in 1/200 diluted antibody against vimentin (MAB3400, Millipore) or *β*-tubulin (MAB3408, Millipore), respectively, for 1 h, followed by incubation with a 200 times dilution of their respective secondary antibodies (A11003 and A11059, Life Technologies) for 1 h. For *α*-SMA staining, 1/500 diluted antibody against *α*-SMA (A2547, Sigma-Aldrich) and 1/500 diluted secondary antibody (Alexa Fluor 546 rabbit anti-mouse, A11060, Invitrogen) were used.

The cells were observed with an inverted fluorescence microscope (IX71, Olympus) equipped with a charge-coupled device (Abrio-LS, CRi) through an objective lens of 60× (UPLSAPO60XW, NA = 1.20, Olympus; UPLSAPO60XS2, Olympus) or 30× (UPLSAPO30XS, Olympus). Retardation and fluorescence images were captured. For *α*-SMA staining cells, DIC images were also captured. The cell region was defined in DIC images by manually outlining the cell, and the area, *A*_*Cell*_, of the cell region was measured. By transferring the cell outline to the fluorescence image, an average grey value of *α*-SMA, *I*_*Cell*_, within the cell was obtained. Similarly, average cell retardation, *Ret*_*Cell*_, within the cell was also obtained. A series of image analyses was performed with image analysis software (ImageJ 1.47 h, National Institutes of Health).

### Regulation of contractile forces

After being cultured on a glass-bottom dish for 60–72 h, VSMCs were treated with normal medium supplemented with 10 nM calyculin A (032–14451, Wako Pure Chemicals) to induce actomyosin contraction^28^ or 20 µM Y-27632 (257-00511, Wako Pure Chemicals) to inhibit actomyosin contraction^41^. Images of phase contrast and retardation were captured every 10 min for 50 min after the treatment. At each time point, the cell area, *A*_*Cell*_, was measured by manually outlining the shape of the VSMCs in the phase contrast images. Then, the cells were selected in the retardation image, and the spatially averaged cell retardation, *Ret*_*Cell*_, was calculated. To consider the changes in the cell area due to the administration of contraction and relaxation reagents, the total cell retardation, *Ret*_*CellTotal*_, was obtained by multiplying the averaged cell retardation, *Ret*_*Cell*_, by the cell area, *A*_*Cell*_. The average and total cell retardations were normalized by the values before treatment in each cell.

### Traction force microscopy

Traction force microscopy with some modifications of Lee *et al*.^14^ was performed. To promote adhesion of the gel to the glass, a 35 mm-diameter glass-bottom dish was incubated in 0.1 M NaOH for 1 h with 1% (3-aminopropyl) triethoxysilane (APTES, A3648-100ML, Sigma-Aldrich) for 2 h, and the dish was dried. Polyacrylamide (PAA) gel was prepared as 9% of the total monomer concentration and 0.5% of cross-linkers by mixing acrylamide-bis solution (40% Acrylamide/Bis solution 19:1, Bio-Rad), 40% acrylamide (40% Acrylamide, Bio-Rad), photoactive cross-linker (final 1 mg/mL, Irgacure2959, Ciba), and distilled water. By applying 9 µL of the monomer and cross-linker solutions, the thickness of the PAA gel was set at 30 µm on a glass-bottom dish coated with amino silane. Fluorescent microspheres (*ϕ* = 0.2 µm, F-8811, Thermo Fisher Scientific) were fixed on an 18 × 18 mm coverslip, and the coverslip was placed on the PAA solution as a top cover. Then, the PAA solution was cross-linked by exposure to ultraviolet light (wavelength = 365 nm) for 20 min. After the removal of the coverslip, 200 µL of 1 mM sulfosuccinimidyl 6-(4′-azido-2′-nitrophenylamino) hexanoate (Sulfo-SANPAH, 22589, Thermo Fisher Scientific) in 50 mM HEPES (345-06681, Chemical Dojin) was dropped on the PAA gel, and ultraviolet light was applied for 15 min, followed by washing with HEPES twice. Finally, the PAA gel was immersed in 10% fibronectin in PBS for 2 h and medium for 1 h to exchange the solution in the gel. The pipette aspiration method^42^ results showed that the elastic modulus of the gel was 16 kPa.

VSMCs were cultured on the PAA gels for 60–72 h and observed under the fluorescence microscope equipped with the birefringence imaging system at 37 °C in an atmosphere of 5% carbon dioxide. After retardation of the VSMCs was imaged, images of fluorescent microspheres (*ϕ* = 0.2 µm, F-8811, Thermo Fisher Scientific) were captured through a 60× water immersion objective lens (UPLSAPO60XW, NA = 1.20, Olympus) and a mirror unit (U-MNIBA3, Olympus). DIC images of VSMCs were also captured. Then, the VSMCs were removed from the gel in 0.05% Trypsin-EDTA (25200, Gibco), and images of the fluorescent microsphere were captured again.

Image analysis software (ImageJ 1.47 h) was used to estimate CTF. The displacement field between the fluorescent microsphere images before and after the removal of VSMCs was obtained by positional correction^40^ and repeated particle image velocimetry^43^. The cell traction stress was determined from the displacement field with Fourier transform traction force cytometry^44^. The cell traction stress was divided into components corresponding to the major and minor axes of a cell, which were determined from the best-fit ellipse for each cell. Only the stress components in the positive direction of the major axis near the cell area (approximately 10 µm from the cell area) were summed. Similarly, those in the minor axis direction were summed. Then, the root mean square of the stress along the major and minor axes was calculated to obtain the total cell traction force, *CTF*_*CellTotal*_, of a single cell. The total cell traction force, *CTF*_*CellTotal*_, was compared with the total cell retardation, *Ret*_*CellTotal*_, which was obtained as stated in the “Regulation of contractile forces” section.

### Assessment of local retardation

A cell was divided into a central region and edge regions to evaluate the spatial distribution of retardation within a VSMC. After obtaining the best-fit ellipse to the cell, four lines were equidistantly drawn perpendicularly to the major axis of the ellipse to divide the cell into five regions. The regions at both edges of the cell were defined as the edge region, whereas the remaining three regions were defined as the central region.

### Statistics

The data are presented as the mean ± SD. The Mann-Whitney U test was used to identify differences between two groups. A difference was considered statistically significant for *p* < 0.05 in all tests. Changes in the retardation of cells treated with contraction and relaxation chemicals against controls were evaluated at each time point using Steel’s test. A correlation between two data points was evaluated with Pearson’s correlation coefficient. The numbers *N* and *n* represent the number of dishes and cells, respectively.

## Supplementary information

Movie 1

Movie 2

Movie 3

Supplemental Information
