# Supplementary material for: Photoelasticity-based evaluation of cellular contractile force for phenotypic discrimination of vascular smooth muscle cells
Source: Sci Rep. 2019 Mar 8;9:3960. doi: 10.1038/s41598-019-40578-7 (PMC6408479; doi:10.1038/s41598-019-40578-7)
Supplement: Supplementary file 4 — Supplemental Information [file 41598_2019_40578_MOESM4_ESM.pdf]

Supplementary Information

Photoelasticity-based evaluation of cellular contractile force for phenotypic discrimination of vascular smooth muscle cells

Shukei Sugita[1)]*, Eri Mizutani[2)], Masatoshi Hozaki[1)], Masanori Nakamura[1)], and Takeo Matsumoto[1, 3)], *Nagoya, Japan*

Affiliation:

1. Department of Electrical and Mechanical Engineering, Graduate School of Engineering, Nagoya Institute of Technology
2. Department of Techno-Business Administration, Graduate School of Engineering, Nagoya Institute of Technology
3. Department of Mechanical Systems Engineering, Graduate School of Engineering, Nagoya University (Present)

***To whom correspondence should be addressed:**

Shukei Sugita, Ph.D.

Department of Mechanical Engineering, Graduate School of Engineering, Nagoya Institute of Technology, Gokiso-cho, Showa-ku, Nagoya 466-8555, JAPAN

Tel. and Fax: +81 52 735 7125

E-mail: sugita.shukei@nitech.ac.jp

S1. Fluorescently labelled actin filaments of vascular smooth muscle cells (VSMCs) on a polyacrylamide (PAA) gel

We investigated whether SFs in cells cultured on PAA gels develop as well as those on glass-bottom dishes. The VSMCs on PAA gels were prepared as described in the "Traction force microscopy" section, and actin filaments in the cells were fluorescently labelled with Alexa-Fluor 488 Phalloidin and observed with an inverted fluorescence microscope (IX71, Olympus) equipped with a charge-coupled device (Abrio-LS, CRi) through a 60× objective lens (UPLSAPO60XW, NA = 1.20, Olympus) as described in the "Immunofluorescence microscopy" section. Figure S1 shows typical images of fluorescently labelled actin filaments and the retardation of VSMCs on PAA gels. Actin filaments in VSMCs on PAA gel are more unclear than those on glass-bottom dishes, as shown in Fig. 1. The results show that VSMCs on the PAA gel have fewer and thinner SFs than those on the glass-bottom dish.

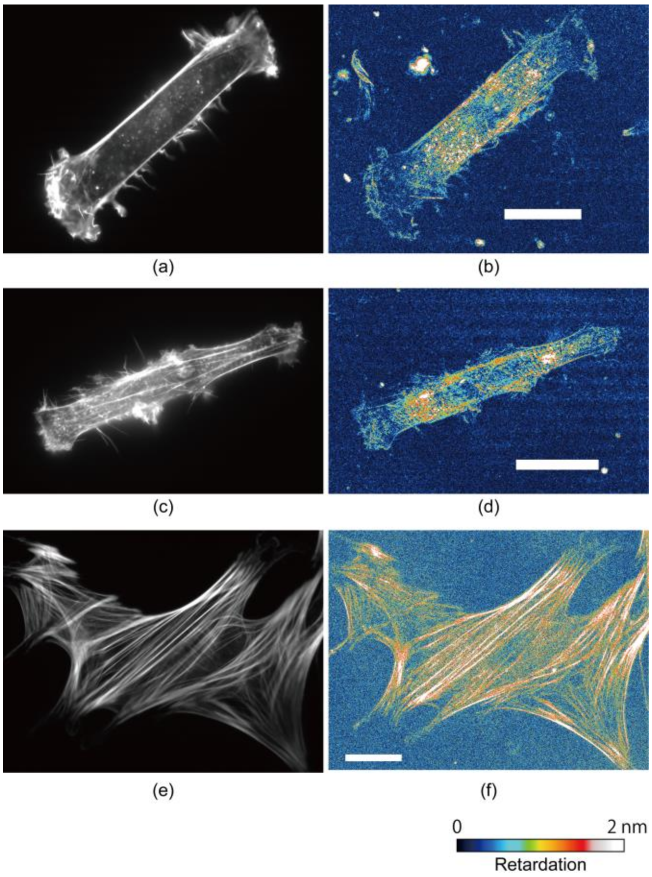

Fig. S1 Typical images of fluorescently labelled actin filaments (a, c, and e) and retardation (b, d, and f) of VSMCs on PAA gels (a–d) and on glass-bottom dishes (e and f). Bars in (b), (d), and (f) = 30 μm.

S2. Retardation of the polyacrylamide (PAA) gel

We investigated whether the PAA gel itself has retardation. PAA gels were fabricated as stated in the “Traction force microscopy” in the Methods section. These gels were sliced into 0.5-mm-thick sections with a micro-slicer (DTK-1000, Dosaka EM). The specimens were cut into a block (width 3 mm× length 20 mm) with a surgical knife. The specimen was stretched with a laboratory-made uniaxial tensile tester[1]. Both ends of the specimen were glued on PET films, and the films were glued on the two lever arms of the tester. The lever arms were mounted on a linear guide with an oppositely threaded screw driven by a stepping motor. Both lever arms were moved by 0.3 mm in 1 s to apply 5% strain to the specimen. At each strain, retardation was imaged under an inverted fluorescence microscope (IX71, Olympus) equipped with a birefringence imaging system (Abrio-LS, CRi) through an objective lens of 2× (PLAPON2X, NA = 0.08, Olympus). Captured images were processed with image analysis software (ImageJ 1.47 h, National Institutes of Health). The average retardation of the PAA gel, Ret_{PAA}, was measured in an area with 4 mm×1.5 mm, and its retardation was compensated by subtracting the retardation in the background area. Figure S2a–d shows typical images of the PAA gel during the uniaxial tensile test. Because the retardation of the gel Ret_{PAA} at strain $\varepsilon = 0$ was not 0 (Fig. S2e), the PAA gel was confirmed to be a birefringent material that has its own retardation. With increased stretch, the retardation of the gel Ret_{PAA} increased (Fig. S2e). These results demonstrate that PAA gels exhibit retardation and retardation changes due to stretching.

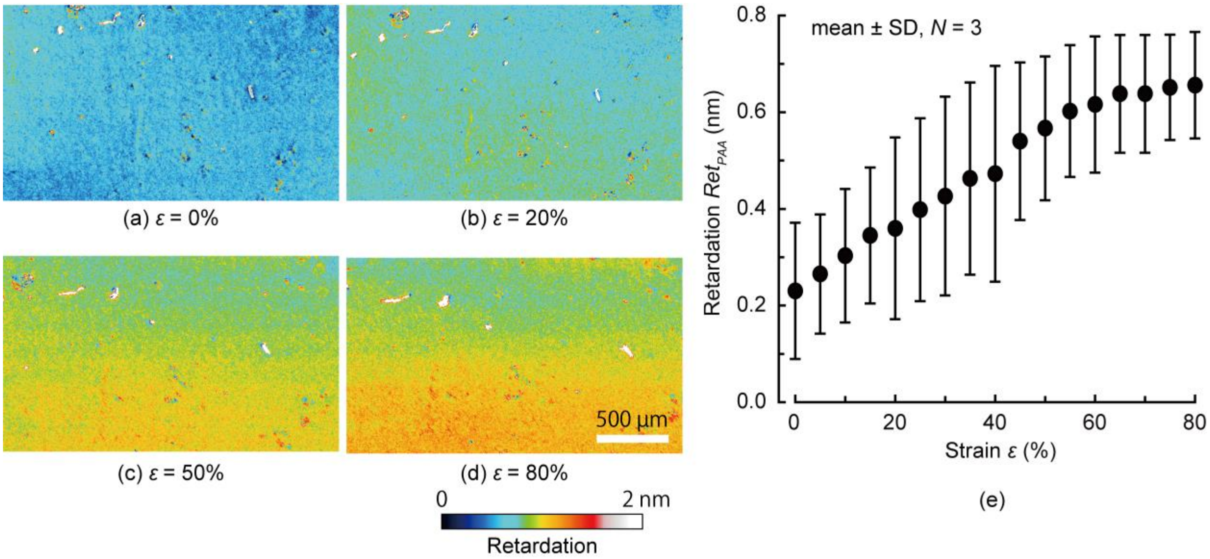

Fig. S2 Retardation of PAA gels. (a–d) Typical image of the PAA gel at strains (a) ε = 0%, (b) ε = 20%, (c) ε = 50%, and (d) ε = 80%. (e) Changes in retardation of the PAA gel Ret_{PAA} during the uniaxial tensile test. *N*, number of PAA gels.

References

1 Sugita, S. & Matsumoto, T. Yielding phenomena of aortic wall and intramural collagen fiber alignment: Possible link to rupture mechanism of aortic aneurysms. *J. Biomech. Sci. Eng.* **8**, 104-113 (2013).

Movie legends

Movie 1

Typical time-lapse images of retardation of a cell after application of DMEM (control).

Movie 2

Typical time-lapse images of retardation of a cell after application of calyculin A.

Movie 3

Typical time-lapse images of retardation of a cell after application of Y-27632.
